# Limited Internal Radiation Exposure Associated with Resettlements to a Radiation-Contaminated Homeland after the Fukushima Daiichi Nuclear Disaster

**DOI:** 10.1371/journal.pone.0081909

**Published:** 2013-12-02

**Authors:** Masaharu Tsubokura, Shigeaki Kato, Masahiko Nihei, Yu Sakuma, Tomoyuki Furutani, Keisuke Uehara, Amina Sugimoto, Shuhei Nomura, Ryugo Hayano, Masahiro Kami, Hajime Watanobe, Yukou Endo

**Affiliations:** 1 Division of Social Communication System for Advanced Clinical Research, Institute of Medical Science, University of Tokyo, Minato-ku, Tokyo, Japan; 2 Department of Radiation Protection, Soma Central Hospital, Soma, Fukushima, Japan; 3 Hirata Radioactivity Inspection Center, Hirata Central Hospital, Hirata, Fukushima, Japan; 4 Faculty of Policy Management, Keio University, Fujisawa, Kanagawa, Japan; 5 Faculty of Environment and Information Studies, Keio University, Fujisawa, Kanazawa, Japan; 6 Department of Global Health Policy, Graduate School of Medicine, University of Tokyo, Bunkyo-ku, Tokyo, Japan; 7 Department of Physics, University of Tokyo, Bunkyo-ku, Tokyo, Japan; 8 Kawauchi Village Mayor, Kawauchi Municipal Government, Kawauchi, Fukushima, Japan; Kagoshima University Graduate School of Medical and Dental Sciences, Japan

## Abstract

Resettlement to their radiation-contaminated hometown could be an option for people displaced at the time of a nuclear disaster; however, little information is available on the safety implications of these resettlement programs. Kawauchi village, located 12–30 km southwest of the Fukushima Daiichi nuclear power plant, was one of the 11 municipalities where mandatory evacuation was ordered by the central government. This village was also the first municipality to organize the return of the villagers. To assess the validity of the Kawauchi villagers’ resettlement program, the levels of internal Cesium (Cs) exposures were comparatively measured in returnees, commuters, and non-returnees among the Kawauchi villagers using a whole body counter. Of 149 individuals, 5 villagers had traceable levels of Cs exposure; the median detected level was 333 Bq/body (range, 309–1050 Bq/kg), and 5.3 Bq/kg (range, 5.1–18.2 Bq/kg). Median annual effective doses of villagers with traceable Cs were 1.1 x 10^-2^ mSv/y (range, 1.0 x 10^-2^-4.1 x 10^-2^ mSv/y). Although returnees had higher chances of consuming locally produced vegetables, Cochran-Mantel-Haenszel test showed that their level of internal radiation exposure was not significantly higher than that in the other 2 groups (p=0.643). The present findings in Kawauchi village imply that it is possible to maintain internal radiation exposure at very low levels even in a highly radiation-contaminated region at the time of a nuclear disaster. Moreover, the risks for internal radiation exposure could be limited with a strict food control intervention after resettlement to the radiation-contaminated village. It is crucial to establish an adequate number of radio-contaminated testing sites within the village, to provide immediate test result feedback to the villagers, and to provide education regarding the importance of re-testing in reducing the risk of high internal radiation exposure.

## Introduction

Radiation exposure can generate long-term risks for disorders such as tumors depending on personal exposure doses[1]. Health threats have emerged in radiation-contaminated areas. Cumulative radiation exposure is a serious public concern in Fukushima, particularly among children, who face greater health risks than adults.

Evacuation from radiation-contaminated areas at the time of a nuclear disaster is an effective strategy to reduce the risks of radiation exposure[2], although there is a risk-benefit trade-off between the long-term effects of low-dose radiation and the serious short-term health effects an evacuation may cause[3]. For instance, the blanket evacuation technique caused a dramatically acute increase in mortality among residents of elderly nursing homes in the Fukushima area[4]. Moreover, evacuation could have long-term adverse health effects; prolonged life as evacuees could represent physical, mental and socioeconomic burdens[5].

Thus, an option for displaced people, who generally have strong affective ties with their place of birth, is resettlement to their radiation-contaminated hometown, as was the case after the Chernobyl disaster[6]. However, little information is available regarding the safety of such resettlement programs to the radiation-contaminated hometowns.

**Figure 1:**
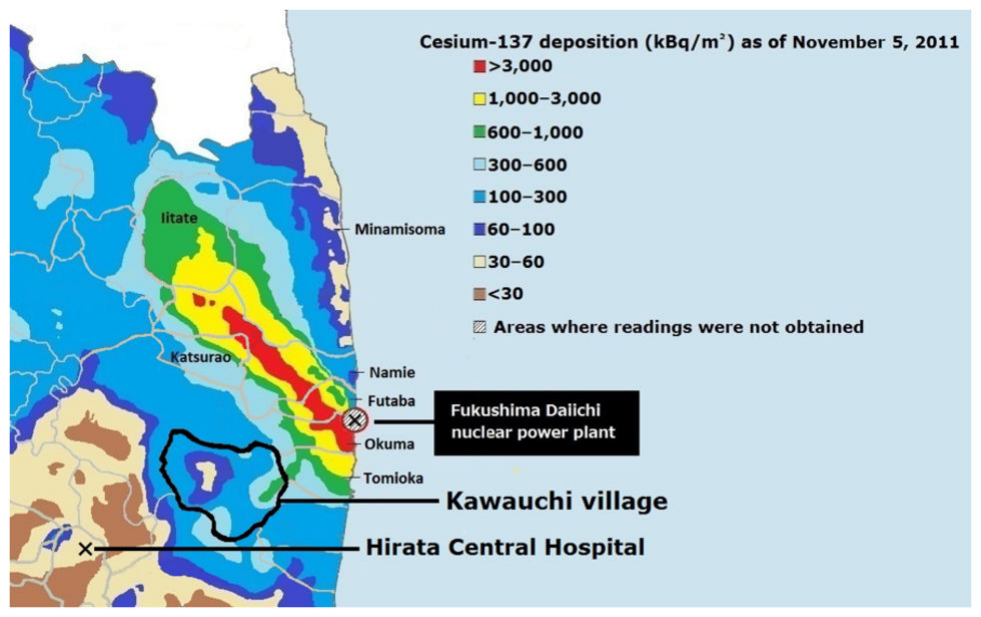
Cesium-137 deposition of soil around Fukushima Daiichi nuclear power plant as of November 5, 2011.

Under such circumstances, the case of Kawauchi village, Fukushima Prefecture, provides useful information concerning the validity of the resettlement program. Kawauchi village is located 12–30 km southwest of the Fukushima Daiichi nuclear power plant. This village was one of the 11 municipalities that were evacuated by central government mandate on March 15, 2011, and it was the first municipality to issue the return of its villagers after the Fukushima Daiichi nuclear power plant disaster[7]. The deposition density of Cesium (Cs)-137 was 43-860 kBq/m^2^ on November 11, 2011 in the Kawauchi area (Figure 1), which was comparable to zone II (555 kBq/m^2^) as of 1988 after the Chernobyl disaster[8,9]. Approximately half a year after the disaster, the central government lifted the emergency evacuation preparation zone in a 20-30 km radius from the Fukushima Daiichi nuclear power plant on September 30, 2011. Taken toghether with the low risk of hydrogen explosion and failure of the cooling system, and the low levels of external radiation exposure measured among the Fukushima residents, the mayor of Kawauchi village issued a declaration for all the former villagers to return home on January 31, 2012[10].

However, many people hesitated to return home, partly owing to the lack of the information on the safety after the resettlement. Elevated air dose levels evoked a feeling of fear of potential irradiation. In addition, primarily because of consumption of potentially radiation-contaminated locally grown food products, a higher level of internal radiation exposure was expected among returnees after their resettlement to Kawauchi village. While the main industry of the region was agriculture, which had a working population share of 55%, the estimated annual effective doses from internal radiation exposure among returnees to the Kawauchi village using the results of the radio-contaminant food testing ranged between 0.37–7.0 mSv/y.[11] High doses from internal radiation exposure happened to be estimated in case of continuous ingestion of highly radio-contaminant food such as mushrooms.

To assess the validity of the Kawauchi villagers’ resettlement program, the levels of internal Cs-137 exposure, known to be representative of total internal radiation exposure[12], were comparatively measured in the returnees and non-returnees among the Kawauchi villagers. In this report, we started off with the presumption that the risk for internal radiation exposure would be limited to returnees. While returnees had higher chances of consuming locally produced vegetables, their level of internal radiation exposure was not significantly higher than that of non-returnees. Based on our findings, we also present and discuss the effective countermeasures to avoid internal contamination among residents after the resettlement, which could be useful for nuclear incidents in the future.

## Materials and Methods

### Ethics approval

Written informed consent was obtained from every participant. The institutional review board of the University of Tokyo approved the study.

### Design, settings, and participants

Kawauchi village has an overall area of 197.4 km^2^ with a population of 2,816 residents prior to the disaster. In order to study the levels of chronic internal radiation exposure among Kawauchi returnees after their return was issued, we reviewed medical records from the radiation exposure screening program conducted at Hirata Central Hospital from April 1, 2012 to March 31, 2013. Major variables represented in the dataset are age, sex, and the total body burden of radioactive Cs (Cs-134 and Cs-137). Internal Cs exposure was measured as both total body exposure and concentration by body weight (Bq/kg).

Starting on November 2011, an extensive voluntary screening program for internal radiation exposure in the form of a medical check-up began at the Hirata Central Hospital, which is located 46.5 km southwest of the plant. All Kawauchi villagers could attend the screening program free of charge. A program notification was sent to each household, including former residents who were evacuated elsewhere but could be tracked using the village’s family registry.

### Whole body counting and internal dose calculation

For the screening program, a whole body counter (WBC) (FASTSCAN 2251, Canberra Industries, Inc., Meriden, USA) was used. All subjects changed into a hospital gown regardless of their surface screening results. The Cs-134 and Cs-137 detection limits were 300 Bq/body with 2-minute scans. The equipment was routinely calibrated as suggested by the manufacturer. Annual effective doses from internal contamination of ingested Cs-134 and Cs-137 were evaluated on the basis of the effective dose coefficients 1.9 x 10^-5^ and 1.3 x 10^-5^ mSv/Bq derived from International Commission on Radiological Protection, Publication 67[13], assuming that the amount of Cs activities detected at the WBC examinations were in an equilibrium state between consecutive ingestion and excretion throughout a year. A person without any recorded exposure, that is under the detection limit, was rated as having exposure of 0 Bq.

### Definition of a returnee (G1), commuter (G2) and non-returnee (G3)

To study any changes in the levels of internal radiation exposure, the villagers were divided into 3 groups: returnees, commuters, and non-returnees. Participants were asked to fill in a questionnaire, which was meant to determine whether he/she returned to the village, or not. The term “returnee (G1)” defined individuals who spent more than 4 days per week at the village. A person who stayed in the village for more than 1 day per week was termed a “commuter (G2)”, and one who did not return to the village at all was termed a “non-returnee (G3)”.

### Data analysis

Within the 3 groups (1), difference in Cs-137 detection rates; and (2) ratio differences in consumption of locally or home-garden produced vegetables were assessed, since the main agricultural commodity of the village was vegetable crops, including potatoes, leafy vegetables, and root vegetables, and rice was banned to crop during the study period. Intake rate of locally produced vegetables was obtained from the questionnaire assigned to each participant at the screening program. The questions on diet asked whether the respondent selected certain produce based on its origin from the supermarket, or simply used local farms. We use the detection rate of Cs-137 (not Cs-134) since the amount of Cs-134 depends on the time of the examinations after the disaster due to the relatively shorter half-life of Cs-134 (2years) compared to that of Cs-137 (30years).

For both analysis listed above, Cochran-Mantel-Haenszel test or Fisher’s exact test were applied. P value of less than 0.05 was considered statistically significant. If statistical differences were obtained, a post-hoc power analysis with 80% power was conducted and examined whether the study was appropriately powered.

Analysis 1: Multiple comparisons were conducted on ratio differences between the 3 groups for the proportion of individuals detected with Cs-137.

Analysis 2: Multiple comparisons were conducted on ratio differences between the 3 groups for the proportion of individuals consuming locally grown produce.

## Results

Of the 347 villagers who underwent screening, 10 were excluded owing to their current ambiguous living status (i.e., did or did not return to the village). Thus, 337 individuals were included in the study, of which 149 were returnees, 61 were commuters, and 127 were non-returnees. Table 1 displays the age and sex distribution of the sample. The partial result of an initial analysis based on the same data has already been published[14].

**Table 1:** Number of examinees in the internal radiation exposure screening program.

	**Total**		**G1**		**G2**		**G3**	
	**Number**	**%**	**Number**	**%**	**Number**	**%**	**Number**	**%**
**Age**								
**4 - 12**	14	4.2	5	3.4	3	4.9	6	4.7
**13 - 18**	5	1.5	2	1.3	0	0.0	3	2.4
**19 - 40**	33	9.8	17	11.4	4	6.6	12	9.4
**41 - 60**	90	26.7	34	22.8	26	42.6	30	23.6
**61 - 80**	181	53.7	84	56.4	25	41.0	72	56.7
**81 - 100**	14	4.2	7	4.7	3	4.9	4	3.1
**Total**	337	100	149	100	61	100	127	100
**Female**	189	56.1	77	51.7	30	49.2	82	64.6

### Levels of internal radiation exposure amongst the returnees

Of 149 returnees who attended the screening program, the mean age of the sample was 60.7 years. While 1,299 of 2,816 individuals returned to the village by May 2013, 11.5% of returnees were included in this study. The dataset well represents the actual returnees in terms of age and sex. Of 149 individuals, 5 villagers had traceable levels of Cs (Cs-134 and Cs-137) and the median detected level was 333 Bq/body (range, 309 - 1050 Bq/kg), and 5.3 Bq/kg (range, 5.1 - 18.2 Bq/kg).

Median annual effective dose of villagers with traceable Cs was 1.1 x 10^-2^ mSv/y (range, 1.0 x 10^-2^ – 4.1 x 10^-2^ mSv/y). Cs was not detected among most of the participants (96.6 %). No radionuclide other than Cs and Potassium-40 was detected. 

### Statistical Analysis

#### Differences in the detection rates of Cs-137 among the 3 groups

**Figure 2:**
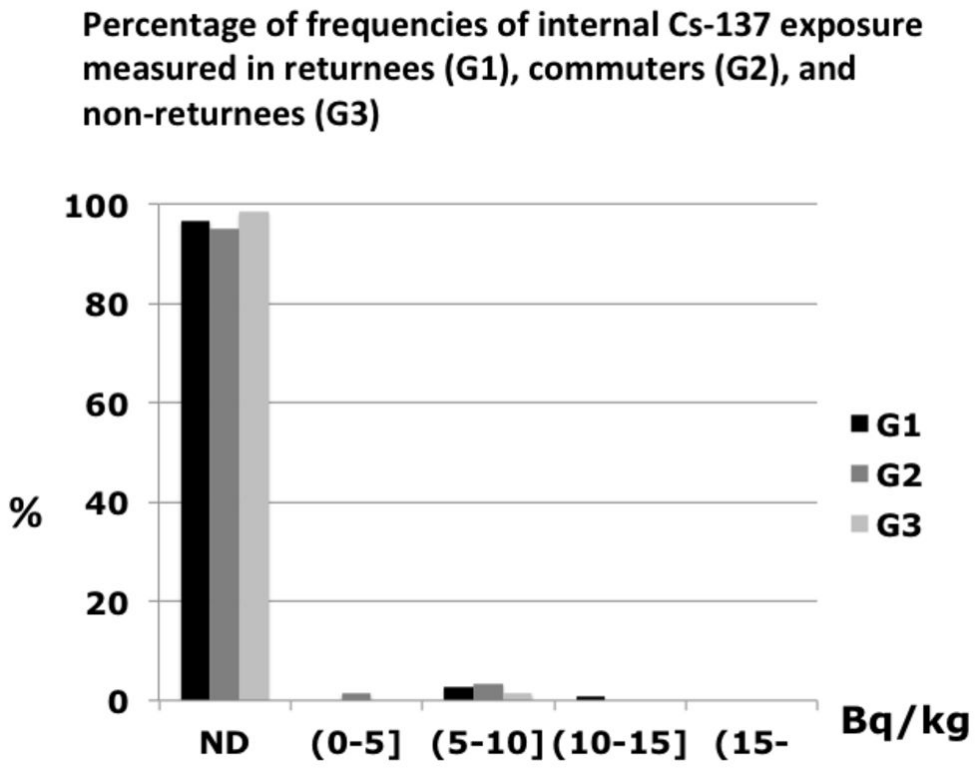
Percentage of frequencies of internal Cs-137 exposure measured in returnees (G1), commuters (G2), and non-returnees (G3) in 5 Bq/kg increments, the numbers of individuals included are 149 returnees, 61 commuters and 127 non-returnees. ND: not detected.

Of 337 individuals, 10 villagers had internal radiation exposure above the detection limit, and the levels of internal Cs-137 ranged from 309 to 629 Bq/body (4.5-10.9 Bq/kg). (Table 2) Median annual effective dose among villagers with detected Cs-137 was calculated as 1.2 x 10^-2^ mSv/y. Figure 2 shows the levels of internal radiation exposure of Cs-137 detected among the 3 groups (G1, G2 and G3). The proportions of individuals with internal radiation exposure above the detection limit were 3.4%, 1.6% and 3.1% for G1, G2, and G3, respectively. (Table 3) Cochran-Mantel-Haenzel test showed that there was no statistically significant difference in the detection rates among the 3 groups (p=0.643). Table 4 showed proportions of residents with internal radiation exposure above the detection limit by sex and age group. Fisher’s exact test showed that there were no statistically significant differences in the detection rates by sex and age.

**Table 2:** List of subjects with traceable levels of Cs in Kawauchi village.

			**Cs-134**		**Cs-137**	
**Age**	**Sex**	**Group**	**Bq/body**	**Bq/kg**	**Bq/body**	**Bq/kg**
63	male	G1	ND	ND	349	5.53
63	male	G1	ND	ND	333	5.30
66	male	G1	421	7.31	629	10.92
69	male	G1	ND	ND	313	5.10
74	male	G1	ND	ND	309	5.10
67	male	G2	409	5.30	513	6.60
61	male	G3	ND	ND	433	6.42
61	male	G3	ND	ND	611	9.55
63	male	G3	ND	ND	380	4.48
66	female	G3	308	5.28	322	5.52

**Table 3:** Proportion of residents with internal radiation exposure of Cs-137 above the detection limit by population group.

**Group**	**Proportion (%)**	**95%CI (%)**	
**G1**	3.4	1.1 to 7.7	
**G2**	1.6	0.0 to 8.8	p=0.643*
**G3**	3.1	0.9 to 7.9	

* Cochran-Mantel-Haenszel test was performed to examine the difference of detection rates among 3 groups.

**Table 4:** Proportion of residents with internal radiation exposure of Cs-137 above the detection limit by sex and age.

**Sex**	**Male**		**Female**		**Total**	
**Age**	**Proportion (%)**	**95%CI (%)**	**Proportion (%)**	**95%CI (%)**	**Proportion (%)**	**95%CI (%)**
**4-12**	0.0	0.0 to 30.8	0.0	0.0 to 60.2	0.0	0.0 to 10.3
**13-18**	-	-	0.0	0.0 to 52.2	0.0	0.0 to 52.2
**19-40**	0.0	0.0 to 20.6	0.0	0.0 to 18.5	0.0	0.0 to 10.3
**41-60**	0.0	0.0 to 11.2	0.0	0.0 to 5.9	0.0	0.0 to 3.9
**61-80**	11.0	4.8 to 18.7	1.0	0.0 to 5.4	5.5	2.7 to 9.9
**81-100**	0.0	0.0 to 30.8	0.0	0.0 to 60.2	0.0	0.0 to 23.2
**Total**	6.1	2.7 to 10.8	0.5	0.0 to 2.9	3.0	1.4 to 5.2

#### Comparisons of ratio differences between the 3 groups for the proportion of individuals consuming locally grown produce

A comparison on the amounts of Fukushima produced and non-Fukushima produced vegetable consumption was made between the 3 groups. Each group had 43.2%, 16.1%, and 21.3% of individuals eating locally produced vegetables, respectively. (Table 5) There was a significant difference in the proportion of individuals consuming locally grown products (p=0.0018). Fisher’s exact test showed that there were no statistically significant differences in the proportion of individuals consuming locally grown products by sex and age. A post-hoc power analysis showed that the power of these tests were 85.3% and 86.9%, respectively.

**Table 5:** Proportions of residents consuming locally grown products by sex, age and population group.

	**Sex**	**Male**		**Female**		**Total**	
**Group**	**Age**	**Proportion (%)**	**95%CI (%)**	**Proportion (%)**	**95%CI (%)**	**Proportion (%)**	**95%CI (%)**
**G1**	4-12	0.0	0.0 to 97.5	0.0	0.0 to 84.2	0.0	0.0 to 70.8
	13-18	-	-	0.0	0.0 to 97.5	0.0	0.0 to 97.5
	19-40	20.0	0.5 to 71.6	0.0	0.0 to 60.2	11.1	0.3 to 48.2
	41-60	41.7	15.2 to 72.3	70.0	34.8 to 93.3	54.5	32.2 to 75.6
	61-80	42.9	21.8 to 66.0	50.0	29.9 to 70.1	46.8	32.1 to 61.9
	81-100	60.0	14.7 to 94.7	0.0	0.0 to 97.5	50.0	11.8 to 88.2
	Total	40.9	26.3 to 56.8	45.5	30.4 to 61.2	43.2	32.7 to 54.2
**G2**	4-12	0.0	0.0 to 97.5	-	-	0.0	0.0 to 97.5
	13-18	-	-	-	-	-	-
	19-40	50.0	1.3 to 98.7	0.0	0.0 to 97.5	33.3	0.8 to 90.6
	41-60	0.0	0.0 to 45.9	14.3	0.4 to 57.9	7.7	0.2 to 36.0
	61-80	20.0	0.5 to 71.6	33.3	4.3 to 77.7	27.3	6.0 to 61.0
	81-100	0.0	0.0 to 84.2	0.0	0.0 to 97.5	0.0	0.0 to 70.8
	Total	12.5	1.6 to 38.3	20.0	4.3 to 48.1	16.1	5.5 to 33.7
**G3**	4-12	0.0	0.0 to 60.2	0.0	0.0 to 84.2	0.0	0.0 to 45.9
	13-18	-	-	33.3	0.8 to 90.6	33.3	0.8 to 90.6
	19-40	33.3	0.8 to 90.6	0.0	0.0 to 45.9	11.1	0.3 to 48.2
	41-60	33.3	0.8 to 90.6	31.3	11.0 to 58.7	31.6	12.6 to 56.6
	61-80	16.7	3.6 to 41.4	18.2	5.2 to 40.3	17.5	7.3 to 32.8
	81-100	50.0	1.3 to 98.7	100.0	2.5 to 100.0	66.7	9.4 to 99.2
	Total	20.0	7.7 to 38.6	22.0	11.5 to 36.0	21.3	12.9 to 31.8

## Discussion

Resettlement to their radiation-contaminated hometown could be an option for displaced people after a nuclear disaster; however, it is difficult to determine the most suitable time span needed prior to the resettlement. Although a higher level of internal radiation exposure was expected among Kawauchi village returnees owing primarily to consumption of potentially radiation-contaminated, locally grown produce, the validity of the Kawauchi villagers’ resettlement program had yet to be assessed until now.

This study reveals that the measured levels of internal radiation exposure were low among the Kawauchi villagers, even among the returnees. While the detected levels of internal radiation exposure were comparable to those living near the damaged nuclear power plant[15], the countermeasures to avoid internal contamination taken in the Kawauchi village appeared to be successful so far after the resettlement. Although ambient dose of Kawauchi village was relatively low compared with other areas around the damaged nuclear power plant, the results could imply that it is possible to maintain internal radiation exposure at very low levels even in a radiation-contaminated region after a nuclear disaster. While the distribution of internal Cs-137 exposure shown in Figure 2 appears to be bi-modal, this is partly because a person without any recorded exposure, that is under the detection limit, was rated as having exposure of 0 Bq.

This study indicated that despite the returnees being more likely to consume locally produced vegetables, their level of internal radiation exposure was not significantly higher than that in the other 2 groups. We can attribute the well-controlled, low level of internal radiation exposure observed in the Kawauchi village returnees despite the increased consumption of locally grown produce to a few measures taken. Firstly, food contaminant testing has been well regulated in this area. It is well known that an intake of heavily radiation-contaminated food products is the main cause of chronic internal radiation exposure around Chernobyl[16]. At Kawauchi village, there are 7 testing locations including the local nursery, the elementary and junior high schools, and the health-care center, where 2,655 tests were conducted during 2012. All the villagers were notified about the test results, and were informed to strictly restrain their intake of contaminated foods. Repetition of the test was considered of great importance, and the test results were used to raise awareness among the villagers. Therefore, we consider that when a municipality decides to allow the return of villagers, it is crucial to establish an adequate number of radio-contaminant testing sites within the village, to provide immediate feedback of the test results to the villagers, as well as to educate them on the importance of repeating the same test and how it enables a reduction in the risk of high internal radiation exposure.

Secondly, the main agricultural products in the village, such as potatoes, leafy vegetables, and root vegetables, will generally absorb a low level of radiation-contamination because of their low plant-soil concentration factors to transfer efficiencies of radioactive materials from soil to plant[17]. The major pathways of Cs contamination from the soil to food chains and to the local population is the regular intake of local grown produce with high plant-soil concentration factors such as mushrooms and wild berries[16]. In contrast, consumption of agricultural products such as potatoes, garden fruits, among other vegetables do not contribute significantly to the daily Cs intake of the inhabitants[18]. Further, mushrooms and mountain vegetables tend to have higher Cs contamination levels of above 100 Bq/kg in comparison with the rest of the food products tested according to the results reported by food monitoring agents in Kawauchi village[19]. Different types of foods grown will behave differently to the levels of radiation contamination in the soil. Hence, when a municipality allows the return of villagers, it is important to identify the major agricultural produce in the area and its potential plant-soil concentration factors. If plant-soil concentration factors of locally grown produce are low, the risk of internal radiation exposure will also tend to be low. In the event of a high plant-soil concentration factor, more attention is needed to avoid extra internal radiation exposure by continuous monitoring of radioactive levels in potentially contaminated products.

Additionally, local clinicians also need to be aware that too much concern over a product transfer coefficient may indirectly and negatively affect the nutritional and life balance of an individual. In the near future, it is important to not only focus on reducing the risk of radiation exposure, but also to care for the other chronic illnesses the villagers may present including hypertension, hyperlipidemia, osteoporosis, and diabetes, as we reported[20-22], and is generally handled in other regions in Japan after the disaster[23,24]. The deterioration of chronic diseases might be associated with changes of life-style attempting to reduce radiation exposures.

An average annual effective dose of internal radiation exposure of a Kawauchi villager, according to soil and food contamination data, was estimated between 0.37–7.0 mSv/y[11], and high doses from internal radiation exposure happened to be estimated in case of continuous ingestion of highly radio-contaminant food such as mushrooms. However, this study indicates that the annual effective dose from ingested Cs among them was below 2.5 x 10^-2^ mSv/y approximately, assuming that the amount of Cs-134 and Cs-137 activities were in an equilibrium state of the detection limits of the examinations (i.e. 300 Bq/body). This result demonstrated that levels of chronic internal radiation exposure in Kawauchi villagers were limited, and the actual measured level of internal radiation exposure was lower than that estimated in this study. Therefore, the estimation of the risk assessment and determination of the appropriate time span to allow the return of villagers to a radiation-contaminated village on the basis of estimated internal radiation exposure alone might not be sufficient or appropriate. It is known that estimated levels of internal radiation exposure were often overestimated in the case of the Chernobyl disaster[25]. While the results of dose reconstruction sometimes do not correspond to those of the actual measurements[26], the results of the present study indicate a possibility to overestimate the levels of internal radiation exposure if only the data on soil and food contamination are used.

There are a few limitations to this study. Firstly, subject screening was conducted soon after the villagers’ return; therefore, more time may have been needed for radionuclides to accumulate in the body. Secondly, there is a high probability of sample selection bias because the sample size was small, mainly owing to the small size of the whole village population, and it is possible that individuals more concerned with the radiation contamination came to the screening program.

## Conclusion

The present study concludes that the risks for internal radiation exposure could be limited after the resettlement of the villagers to their village with a strict food control intervention.
